# Alarm Management in Provisional COVID-19 Intensive Care Units: Retrospective Analysis and Recommendations for Future Pandemics

**DOI:** 10.2196/58347

**Published:** 2024-09-09

**Authors:** Maximilian Markus Wunderlich, Nicolas Frey, Sandro Amende-Wolf, Carl Hinrichs, Felix Balzer, Akira-Sebastian Poncette

**Affiliations:** 1 Institute of Medical Informatics Charité – Universitätsmedizin Berlin Corporate Member of Freie Universität Berlin, Humboldt-Universität zu Berlin, and Berlin Institute of Health Berlin Germany; 2 Department of Nephrology and Medical Intensive Care Charité – Universitätsmedizin Berlin Corporate Member of Freie Universität Berlin, Humboldt-Universität zu Berlin, and Berlin Institute of Health Berlin Germany

**Keywords:** patient monitoring, intensive care unit, ICU, alarm fatigue, alarm management, patient safety, alarm system, alarm system quality, medical devices, clinical alarms, COVID-19

## Abstract

**Background:**

In response to the high patient admission rates during the COVID-19 pandemic, provisional intensive care units (ICUs) were set up, equipped with temporary monitoring and alarm systems. We sought to find out whether the provisional ICU setting led to a higher alarm burden and more staff with alarm fatigue.

**Objective:**

We aimed to compare alarm situations between provisional COVID-19 ICUs and non–COVID-19 ICUs during the second COVID-19 wave in Berlin, Germany. The study focused on measuring alarms per bed per day, identifying medical devices with higher alarm frequencies in COVID-19 settings, evaluating the median duration of alarms in both types of ICUs, and assessing the level of alarm fatigue experienced by health care staff.

**Methods:**

Our approach involved a comparative analysis of alarm data from 2 provisional COVID-19 ICUs and 2 standard non–COVID-19 ICUs. Through interviews with medical experts, we formulated hypotheses about potential differences in alarm load, alarm duration, alarm types, and staff alarm fatigue between the 2 ICU types. We analyzed alarm log data from the patient monitoring systems of all 4 ICUs to inferentially assess the differences. In addition, we assessed staff alarm fatigue with a questionnaire, aiming to comprehensively understand the impact of the alarm situation on health care personnel.

**Results:**

COVID-19 ICUs had significantly more alarms per bed per day than non–COVID-19 ICUs (*P*<.001), and the majority of the staff lacked experience with the alarm system. The overall median alarm duration was similar in both ICU types. We found no COVID-19–specific alarm patterns. The alarm fatigue questionnaire results suggest that staff in both types of ICUs experienced alarm fatigue. However, physicians and nurses who were working in COVID-19 ICUs reported a significantly higher level of alarm fatigue (*P*=.04).

**Conclusions:**

Staff in COVID-19 ICUs were exposed to a higher alarm load, and the majority lacked experience with alarm management and the alarm system. We recommend training and educating ICU staff in alarm management, emphasizing the importance of alarm management training as part of the preparations for future pandemics. However, the limitations of our study design and the specific pandemic conditions warrant further studies to confirm these findings and to explore effective alarm management strategies in different ICU settings.

## Introduction

### Background

Patients critically ill with COVID-19 frequently experience multiple organ failure and need life-sustaining measures such as continuous renal replacement therapy, mechanical ventilation, or even extracorporeal membrane oxygenation [1]. Therefore, during major COVID-19 outbreaks (eg, in 2019 and 2020), intensive care units (ICUs) worldwide saw an increase in admission rates of up to 300% [2]. Intensive care capacities became stretched; in some regions, treatment facilities were fully occupied [3]. Hospitals thus had to increase their intensive care capacities to cope with the proliferating number of patients with COVID-19 [4]. As a consequence, in many countries, provisional ICUs were set up [5-9].

In provisional COVID-19 ICUs, as in standard ICU settings, patients require continuous monitoring of their vital signs, which include heart rate, cardiac rhythm, and peripheral oxygen saturation (SpO_2_) via pulse oximetry. If an abnormal situation occurs (eg, a vital sign exceeds a predetermined threshold), an alarm is issued to alert clinicians [10]. However, ICU staff today often experience alarm fatigue [11] because of high rates of false positive or nonactionable alarms [12]. Alarm fatigue can be hazardous for patient safety, especially when critical alarms are missed [13,14]. The constant noise can cause high levels of stress among staff and disrupt patients’ sleep-wake cycles, increasing the risk of delirium and potentially poorer recovery outcomes [15,16].

In COVID-19 ICUs, the alarm situation might have been even worse: ICU staff, likely overworked due to an overwhelming patient load and a massive workload, had to cope with the psychological burden of the fear of infection and the distressing reality of witnessing numerous patient deaths. ICU staff experienced poor mental health during the COVID-19 pandemic [17-19], and more errors in clinical settings occurred [20].

Moreover, the makeshift nature of the ICUs, which were set up in haste to handle the surge in patients, likely exacerbated these problems. The often improvised and provisional nature of these ICUs likely introduced additional operational complexities and stressors, such as limited space and insufficient technical equipment, further taxing the already strained staff. All these issues may have been compounded by a loud and potentially unreliable alarm system.

### Objectives

This study systematically assesses the alarm situations in 2 provisional COVID-19 ICUs and 2 non–COVID-19 ICUs, focusing on both the actual alarm rates and the extent of alarm fatigue experienced by ICU staff. By incorporating expert interviews with medical professionals who have worked in COVID-19 ICUs, alongside quantitative analyses of alarm log data from the bedside monitors [21-23] and results from the alarm fatigue questionnaire [24], this investigation follows a multimodal approach.

## Methods

### Study Design

In this study, the alarm situations in 2 provisional COVID-19 ICUs were compared with those in 2 non–COVID-19 ICUs; all 4 ICUs were situated on the same campus at a tertiary care university hospital in Berlin, Germany. A retrospective observational analytical study design was used, with the comparison being approached from three perspectives: (1) hypotheses were derived and tested based on interviews with medical experts, exploring potential differences in alarm situations between COVID-19 and non–COVID-19 ICUs; (2) alarm log data from both types of ICUs were analyzed; and (3) the results from an alarm fatigue questionnaire, which was administered as part of another study [24] during the same period, were collected and analyzed.

The non–COVID-19 ICUs consist of an interdisciplinary ICU with patients who have recently undergone surgery or are in the perioperative stage (hereinafter *surgical ICU*); and an ICU with a focus on internal medicine (hereinafter *medical ICU*) where patients with severe infection, cardiac diseases, kidney failure, or other single- or multiple-organ failures are treated.

The medical and surgical ICUs have single and multiple bedrooms and contain 20 and 21 beds, respectively. The COVID-19 ICU was divided into 2 separate units: COVID-19 ICU-A had single bedrooms with 16 beds, and COVID-19 ICU-B had single and multiple bedrooms with 24 beds.

### Expert Interviews for Hypothesis Design

To derive hypotheses regarding how alarm situations might differ between COVID-19 and non–COVID-19 ICUs, 5 medical experts (physicians: n=2, 40%; nurses: n=3, 60%) were interviewed from July to December 2021. The interviews were semistructured [25,26]. The foundational questions were grounded in our overarching research question: “What distinguishes the alarm situations in COVID-19 ICUs from those in non–COVID-19 ICUs?” A detailed list of these questions can be found in Table S1 in Multimedia Appendix 1.

In addition, 2 job shadowing sessions were conducted (one on October 1, 2021, in the medical ICU; and another on November 12, 2021, in COVID-19 ICU-B). During the second session, an interview was conducted in COVID-19 ICU-B. The remaining 4 interviews were held online via Microsoft Teams [27]. An integral aspect of the interviews was that beyond the structured questions, the medical experts were proactively asked about potential hypotheses regarding the differences in alarm situations between the ICU types.

Subsequent to the interviews, the collected data, including transcripts and notes, were meticulously examined. The hypotheses were derived through an in-depth examination and collective discussion within an interdisciplinary team.

### Statistical Analysis

#### Data Analysis

The alarm logs were processed and analyzed as previously described [22], using R (R Foundation for Statistical Computing) [28] with RStudio (Posit Software, PBC) [29] in combination with the following packages: *lubridate* [30], *ggplot2* [31], and *dplyr* [32]. The logs were extracted from the Philips IntelliVue patient monitoring systems (in the surgical and medical ICUs: MX800, software version *m*; in the COVID-19 ICUs: MX750, software version *p*) as CSV files from the non–COVID-19 ICUs and as XML files from the COVID-19 ICUs (structure of the cleaned alarm log data frames is listed in Table S2 Multimedia Appendix 1). Alarm signals from mechanical ventilation devices were not correctly stored in the alarm log data due to transmission errors and were therefore excluded from the analysis. The included alarm signals and the assignment to their medical device are listed in Table S3 in Multimedia Appendix 1. The logs range across 113 days (from November 19, 2020, to March 11, 2021).

#### Hypothesis Testing

To test for statistical differences between COVID-19 and non–COVID-19 ICUs, the units were grouped accordingly. All tests were 1-tailed, with a significance level of α=.05. *P* values were adjusted using the Bonferroni correction. The first hypothesis (H1) proposed that the alarm load is higher in COVID-19 ICUs than in non–COVID-19 ICUs. This hypothesis was further subdivided into total alarm load, clinical red alarms, clinical yellow alarms, and technical alarms.

The second hypothesis (H2) posited that more alarms are issued from specific medical devices, namely electrocardiogram (ECG) and invasive blood pressure (IBP) devices, in COVID-19 ICUs than in non–COVID-19 ICUs. The alarms from these devices were further categorized based on the alarm color (red: potentially life-threatening events; yellow: vital signs exceed predetermined thresholds).

The third hypothesis (H3) proposed that the alarm duration is longer in COVID-19 ICUs than in non–COVID-19 ICUs. For this hypothesis, the alarm durations from clinical alarms were subdivided into medical devices (non-IBP [NIBP], temperature, SpO_2_, ECG, and IBP devices) and alarm colors (red and yellow).

For H1 and H2, the alarm load was quantified in alarms per bed per day. Given that the distributions were skewed to the right and approximately gamma distributed, a dummy-coded no-intercept generalized linear model with a log link function was used. Cohen *d* was used as the effect size measure and was calculated using the package *effsize* developed by Torchiano [33].

For H3, testing was conducted with median alarm durations. The distributions of alarm durations were highly skewed to the right and approximately exponentially distributed; therefore, nonparametric bootstrapping with the *boot* package developed by Canty et al [34] was used with an a priori estimation of the difference in alarm duration with equation 1.

*H*_0_: *μ*_1_ – *μ*_2_ ≤ 3 **(1)**

*H*_A_: *μ*_1_ – *μ*_2_ > 3

Differences of 3 seconds were considered significant, while smaller effects that could already be significant due to the large sample were considered not significant. A median-based estimator for Cohen *d–*type effect size (equation 2) with an estimation of variance (equation 3) was used, where *k* is the number of units and *l* is the number of alarms.



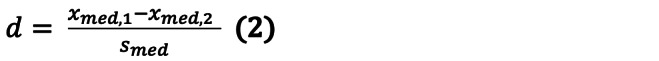





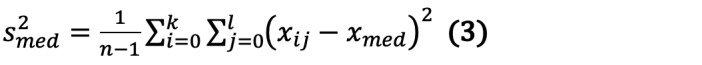



To test the differences in alarm fatigue experienced by ICU staff between COVID-19 and non–COVID-19 ICUs (H4), an unpaired 1-tailed *t* test using Cohen *d* as the effect size measure was conducted.

#### Exploratory Data Analysis

Metrics defined in our previous study [22] were used for the evaluation of alarm situations in the ICUs: alarms per bed per day, critical alarms, alarms per device, alarm flood conditions (≥10 alarms within 10 minutes), use of the alarm pause function per bed per day, proper pause-to-pause ratio, and concurrent alarm duration per bed per day. The last metric was first introduced by Varisco et al [12] and is calculated by summing the number of active parallel alarms. Specifically, if 2 alarms sound simultaneously within a second, this is counted as 1 second of concurrent alarm duration, and if 3 alarms sound simultaneously within a second, 2 seconds are counted.

The metrics were related to bed occupancy and time period to compare the results between the different ICUs. Due to the absence of information regarding the cause of termination in the data, it was not possible to determine the alarm response time. Therefore, alarm duration was used instead.

In the calculations of alarm flood conditions and concurrent alarm duration, only alarms with an auditory modality, specifically yellow and red alarms, were included. For the alarm duration used in calculating concurrent alarm duration, a cutoff was set at 1800 seconds. Alarm durations exceeding this limit were considered outliers and were therefore excluded from the analysis.

#### Alarm Fatigue Questionnaire

The questionnaire data were taken from a separate study [24] that coincided with our data collection phase. The questionnaire, distributed as a web-based survey via REDCap (Research Electronic Data Capture; Vanderbilt University) [35] to ICU staff at the same German hospital between April and June 2021, provided responses from COVID-19 ICU-A, COVID-19 ICU-B, a third COVID-19 ICU (COVID-19 ICU-C), and the 2 non–COVID-19 ICUs (medical and surgical ICUs). The original questionnaire consisted of 27 items; however, only those aligning with the 9-item questionnaire developed by Wunderlich et al [36] were included in this analysis. Each item was measured on a 5-point Likert scale, ranging from *I strongly agree* to *I strongly disagree*.

Demographic questions about work experience, place of work, and position (nurses and physicians, as well as support staff, ie, students or nurses from general wards) were also part of the questionnaire. Only responses from participants who consented to data analysis were included in the study. Submissions with 1 or 2 missing items were imputed at random based on the predictive mean matching algorithm using 1 imputation with the *mice* package [37]. To calculate an alarm fatigue score, the items were scored from –2 (*I strongly disagree*) to 2 (*I strongly agree)*. *I partly agree* was scored with 0. Four items were scored reversely. The sum of all Likert items results in the alarm fatigue score, which ranges from –18 to 18. A score of –18 would indicate that the staff members are not experiencing alarm fatigue at all, while a score of 18 would mean that the staff members are experiencing extreme alarm fatigue; the midpoint is 0. We report the alarm fatigue in percentage as recommended by Wunderlich et al [24] with equation 4.



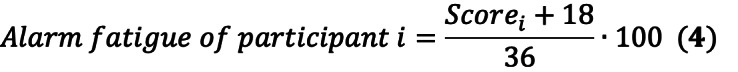



### Ethical Considerations

Ethics approval for this study was granted by the ethics commission of Charité–Universitätsmedizin Berlin (EA4/218/20). All participants provided consent before the study. Data confidentiality was ensured through anonymization in compliance with General Data Protection Regulation. No compensation was provided to participants.

## Results

Beginning with insights from expert interviews to formulate our hypotheses, we proceeded to test them empirically using alarm log data and an alarm fatigue questionnaire, concluding with insights from our exploratory data analysis.

### Expert Interviews for Hypothesis Design

Of the 5 expert interviewees, 2 (40%) reported that only approximately one-third of the staff in COVID-19 ICUs had experience in intensive care, while the remaining two-thirds consisted of nurses, who until then had only worked on general wards; or individuals without specific experience, such as medical students; and even untrained personnel. The staff were assigned different tasks depending on their qualifications. Of the 2 physicians, 1 (50%) suggested that staff were not trained on how to properly apply sensors, such as ECG electrodes, potentially leading to additional (medically irrelevant) alarms. Of the 3 nurses, 1 (33%) suggested that alongside the alarm burden, the high fatality rate among patients imposes psychological strains on the staff. According to the interviewees, the patient cohort in the COVID-19 ICUs presented a more or less homogeneous clinical picture with varying COVID-19 severity. Many patients needed mechanical ventilation and underwent continuous renal replacement therapy with dialysis devices that produce very loud and unpleasant alarms. However, these alarms were not recorded in the alarm logs.

Of the 2 physicians, 1 (50%) reported that patients with COVID-19 infection often have multiple organ failure and a high length of stay; therefore, they are often equipped with ≥7 perfusors for medications (eg, antibiotics, catecholamines, sedatives, and parenteral nutrition), all of which trigger additional alarms that are also not recorded in the alarm logs. All 3 nurses reported a higher alarm burden compared with those working in non–COVID-19 ICUs. All interviewees described the removal and reattachment of all patient sensors during transition from prone to supine position or vice versa as a possible cause of false alarms if the alarm pause function was not used. On the basis of this information, we hypothesized as follows: (H1) The alarm load is higher in COVID-19 ICUs than in non–COVID-19 ICUs.

Of the 3 nurses, 2 (67%) reported that they perceived many patients to be multimorbid, overweight, and of older age, with many having a cardiac or pulmonary history and tachycardia. All interviewees reported that the blood circulation of patients with COVID-19 was extremely unstable, which led us to hypothesize that medical devices related to blood circulation (ie, the ECG, NIBP, or IBP devices) issue more alarms in COVID-19 ICUs: (H2) More alarms are issued from ECG, NIBP, and IBP devices in COVID-19 ICUs than in non–COVID-19 ICUs.

Both COVID-19 ICUs featured long corridors with inward-opening doors, which were usually closed to isolate patients who were infectious. The interviewees reported that the corridors, the dispensary room, and the physician’s room were not equipped with central monitoring; thus, the health care providers had no overview of the patients, which made it difficult to locate the origin of the auditory alarms. Only the nurses’ room was equipped with central monitoring, but alarms could not be turned off remotely. According to the interviewees, temporary arrangements were made by the staff to address this problem, such as leaving the doors to patients’ rooms slightly open or placing speakers linked to the monitors in the hallway. However, to respond to or turn off an alarm, staff had to enter the patient’s room. This required them to don personal protective equipment (ie, gloves, a protective hood, a polypropylene protective gown, and a face shield or goggles), which took approximately 30 seconds and hindered quick movement between rooms. All 3 nurses reported that the protective equipment did not interfere with turning off the alarms, the usability of the monitor displays, or the adjustment of monitor settings. However, they all described it as strenuous and time consuming. Accordingly, we derived the third hypothesis: (H3) The alarm duration is higher in COVID-19 ICUs than in non–COVID-19 ICUs.

Patients in COVID-19 ICUs often present with severe, complex medical conditions that require close monitoring and interventions. Of the 3 nurses, 1 (33%) suggested that next to the alarm burden and heavy workload, the high fatality rate among patients could strain and distress staff psychologically. This mental and emotional strain could potentially affect staff performance and cognitive abilities, potentially hindering their response to alarms. Combining this information with our reasoning for the previous 3 hypotheses, we formulated the fourth hypothesis as follows: (H4) Staff alarm fatigue is higher in COVID-19 ICUs than in non–COVID-19 ICUs.

Having delineated the hypotheses informed by the expert interviews, we proceeded to empirically test each of them, starting with the alarm load in different ICU settings.

### Hypothesis 1: The Alarm Load Is Higher in COVID-19 ICUs Than in Non–COVID-19 ICUs

Significant differences were observed in all 4 tests, which confirmed our hypothesis that the alarm load was higher in provisional COVID-19 ICUs than in non–COVID-19 ICUs (*P*<.001; Tables 1 and 2). COVID-19 ICUs experienced an average of 23% more alarms in total, 41% more red critical alarms, and 24% more yellow alarms compared with non–COVID-19 ICUs. The alarm load caused by technical alarms was 109% higher in COVID-19 ICUs. The alarm load results, subdivided by medical device and alarm color, are reported in Figure S1 in Multimedia Appendix 1. The technical alarm signal that resulted in the most alarms per bed per day was *ECG lead off*, with mean values recorded as follows: surgical 3.42 (SD 2.10), medical 3.84 (SD 2.87), COVID-19 ICU-A 7.22 (SD 1.93), and COVID-19 ICU-B 7.77 (SD 2.41).

**Table 1:** Alarm load from all intensive care units (ICUs; n=113).

	Alarm load (alarms per bed per day)			
	Surgical ICU, mean (SD)	Medical ICU, mean (SD)	COVID-19 ICU-A, mean (SD)	COVID-19 ICU-B, mean (SD)
Total alarms	122.84 (38.95)	122.54 (27.85)	142.47 (41.49)	157.40 (42.58)
Red alarms	10.94 (2.43)	10.99 (2.82)	14.99 (5.89)	15.81 (4.01)
Yellow alarms	108.48 (37.86)	107.71 (26.68)	126.23 (37.93)	140.56 (41.08)
Technical alarms	5.10 (2.17)	5.03 (2.97)	10.30 (2.30)	10.84 (2.51)

**Table 2:** The results from the hypothesis testing for H1 (n=113).

	Alarm load (alarms per bed per day)			*P* value		Cohen *d*
	Non–COVID-19 ICUs^a^, mean (SD)	COVID-19 ICUs, mean (SD)				
Total alarms	122.21 (22.37)	151.26 (31.02)	<.001		1.04	
Red alarms	10.98 (1.90)	15.51 (3.33)	<.001		1.67	
Yellow alarms	108.33 (21.73)	134.59 (29.24)	<.001		1.02	
Technical alarms	5.06 (1.87)	10.62 (1.86)	<.001		2.98	

^a^ICU: intensive care unit.

### Hypothesis 2: More Alarms From ECG, NIBP, and IBP Devices Are Issued in COVID-19 ICUs Than in Non–COVID-19 ICUs

Table 3 shows the results of hypothesis testing for alarms issued by the IBP and ECG devices, subdivided by alarm color and ICU type. In both ICU types, the IBP device was responsible for the majority of the alarms, followed by the ECG and NIBP devices. Yellow alarms issued by the IBP and NIBP devices and red alarms issued by the ECG device occurred significantly more often in COVID-19 ICUs (*P*<.001). However, we did not find significant differences in the occurrence of yellow ECG alarms and red IBP alarms. The results of the alarm load, subdivided by medical device and alarm color, from all ICUs are presented in Table S4 in Multimedia Appendix 1. While certain alarm types exhibited significant differences, the overall impact was not profound enough to affirm the second hypothesis, with the exception of the notable difference in the frequency of red ECG alarms (as indicated by the Cohen *d* value of 1.13).

**Table 3:** Results of the hypothesis testing for H2 (n=113).

Devices	Alarm load (alarms per bed per day)		*P* value	Cohen *d*^a^
	Non–COVID-19 ICUs^b^, mean (SD)	COVID-19 ICUs, mean (SD)		
ECG^c^ (yellow)	39.01 (17.65)	38.79 (18.70)	.99	0.00
ECG (red)	2.51 (1.18)	4.60 (2.34)	<.001	1.13
IBP^d^ (yellow)	53.87 (11.38)	66.16 (18.22)	<.001	0.81
IBP (red)	5.16 (1.14)	5.22 (1.34)	.99	0.05
NIBP^e^	0.34 (0.40)	0.56 (0.51)	<.001	0.49

^a^Cohen *d* has been reported in absolute values.

^b^ICU: intensive care unit.

^c^ECG: electrocardiogram.

^d^IBP: invasive blood pressure.

^e^NIBP: noninvasive blood pressure.

### Hypothesis 3: The Alarm Duration Is Higher in COVID-19 ICUs Than in Non–COVID-19 ICUs

All ICUs had a median clinical alarm duration of 10 seconds. Yellow alarms issued by the NIBP device had the longest alarm durations (Table 4; all group sizes are reported in Table S5 in Multimedia Appendix 1). The durations of yellow alarms triggered by IBP and ECG devices were shorter than those of alarms from all other medical devices, a pattern consistent across all types of ICUs. The median duration of technical alarms in COVID-19 ICUs was significantly longer. Yellow alarms issued by the NIBP, temperature, and SpO_2_ devices had significantly longer durations in COVID-19 ICUs (*P*<.001; Table 5; all group sizes are reported in Table S5 in Multimedia Appendix 1). The results of the median alarm duration, subdivided by medical device and alarm color, are presented in Figure S2 in Multimedia Appendix 1. The results show a mixed picture: <50% of the total alarms had a significant difference in alarm duration between the ICU types, implying that most alarm durations did not differ significantly between the 2 ICU types. As such, our data do not provide sufficient empirical evidence to support H3.

**Table 4:** Results of the hypothesis testing for H3 across each type of intensive care unit (ICU).

	Alarm duration (s)			
	Surgical ICU, median (IQR)	Medical ICU, median (IQR)	COVID-19 ICU-A, median (IQR)	COVID-19 ICU-B, median (IQR)
Clinical alarms	10 (4-27)	10 (4-25)	10 (3-28)	10 (3-29)
Technical alarms	7 (4-66)	4 (4-62)	13 (4-65)	14 (4-68)

**Table 5:** Results of the hypothesis testing for H3 across the 2 types of intensive care units (ICUs).

Alarms	Alarm duration (s)		*P* value	Cohen *d*^a^ type
	Non–COVID-19 ICUs, median (IQR)	COVID-19 ICUs, median (IQR)		
Technical	5 (3-27)	14 (4-67)	<.001	0.016
NIBP^b^	62 (25.00-179.75)	99 (29-337)	<.001	0.094
Temperature	24 (8-86)	32 (9-192)	<.001	0.009
SpO_2_^c^ (yellow)	14 (7-32)	23 (11-57)	<.001	0.069
SpO_2_ (red)	28 (11-89)	33 (14-75)	.11	0.004
IBP^d^ (yellow)	13 (6-32)	11 (4-30)	.99	0.014
IBP (red)	24 (9-84)	20 (9-48)	.99	0.005
ECG^e^ (yellow)	4 (3-9)	3 (2-7)	.99	0.014
ECG (red)	23 (9-119)	16 (8-35)	.99	0.005

^a^Cohen *d* has been reported in absolute values.

^b^NIBP: noninvasive blood pressure.

^c^SpO_2_: peripheral oxygen saturation.

^d^IBP: invasive blood pressure.

^e^ECG: electrocardiogram.

### Hypothesis 4: Staff Alarm Fatigue Is Higher in COVID-19 ICUs Than in Non–COVID-19 ICUs

The questionnaire was completed by 707 participants (n=78, 11% returned blank questionnaires; n=44, 6.2% returned incomplete questionnaires). Of the 585 participants who returned complete questionnaires, we included 144 (24.6%) in the analysis. Of these 144 participants, 88 (61.1%) were from non–COVID-19 ICUs (n=32, 36% from the medical ICU; n=56, 64% from the surgical ICU), and 56 (38.9%) were from COVID-19 ICUs (n=48, 86% from COVID-19 ICU-A; n=8, 14% from COVID-19 ICU-B). The majority of the respondents (92/144, 63.9%) were intensive care nurses (COVID-19 ICUs: 25/56, 45%; non–COVID-19 ICUs: 67/88, 76%). The COVID-19 ICUs had a notable proportion of additional support staff among the respondents, including nursing students and nurses from regular wards (24/56, 43%) compared with non–COVID-19 ICUs (9/88, 10%). The least represented group among the respondents were physicians (COVID-19 ICUs: 7/56, 12%; non–COVID-19 ICUs: 9/88, 10%). The overall alarm fatigue score was higher in COVID-19 ICUs (mean 56.00, SD 15.80) than in non–COVID-19 ICUs (mean 55.27, SD 13.76). Statistical testing of the alarm fatigue score revealed no significant differences between COVID-19 and non–COVID-19 ICUs (t_105.41_=0.2841; *P*=.39; Cohen *d*=0.05). Importantly, when considering only experienced ICU staff—nurses and physicians—the alarm fatigue scores were significantly higher in COVID-19 ICUs than in non–COVID-19 ICUs (t_109_=1.7332; *P*=.04; Cohen *d*=0.363). Figure S3 in Multimedia Appendix 1 depicts the results of the questionnaire, subdivided by profession. Nurses and physicians reported a higher alarm fatigue score than support staff, who generally reported an overall low alarm fatigue score in both ICU types. Given the results of the hypothesis testing (Table 6), we cannot conclusively validate H4, especially when considering all staff types; however, among ICU staff, there is evidence suggesting higher alarm fatigue in COVID-19 ICUs than in non–COVID-19 ICUs.

**Table 6:** Results of the hypothesis testing for H4.

	Alarm fatigue questionnaire scores		*P* value		Cohen *d*
	Non–COVID-19 ICUs, mean (SD)	COVID-19 ICUs, mean (SD)			
All participants	55.27 (13.76)	56.00 (15.80)	.39	0.050	
Clinicians (nurses and physicians)	59.00 (13.05)	64.28 (14.20)	.04	0.363	

### Insights From the Exploratory Data Analysis

While the surgical and medical ICUs were approximately fully occupied over the entire period, the COVID-19 ICUs were often only partly occupied depending on the patient load. Average bed occupancy was 97.26% and 92.27% in the surgical and medical ICUs, respectively; and 88.79% and 82.75% in COVID-19 ICU-A and COVID-19 ICU-B, respectively. Figure S4 in Multimedia Appendix 1 displays the unit occupation over the entire period.

The use of the alarm pause function per bed per day was substantially less frequent in COVID-19 ICUs (mean 5.08, SD 1.69) compared with alarm pauses per bed per day in non–COVID-19 ICUs (mean 12.21, SD 2.21). The medical ICU recorded the highest proper pause-to-pause ratio of 0.08, followed by the surgical ICU and COVID-19 ICU-A, both at 0.04. COVID-19 ICU-B had the lowest pause-to-pause ratio: 0.03. Figure S5 in Multimedia Appendix 1 displays the number of threshold changes per bed per day and profile changes per bed per day from all ICUs.

In COVID-19 ICUs, alarm flood conditions per bed per day occurred on average 35% more frequently (COVID-19 ICU-A: mean 2.60, SD 1.29; COVID-19 ICU-B: mean 2.73, SD 1.47) compared with their non–COVID-19 counterparts (surgical ICU: mean 1.71, SD 1.33; medical ICU: mean 1.95, SD 0.92). In addition, COVID-19 ICUs experienced on average 27% more instances of concurrent alarm duration per bed per day (mean 5201.76, SD 1156.00) than non–COVID-19 ICUs (mean 4101.42, SD 965.00).

## Discussion

### Overview

We compared alarm situations in 2 provisional COVID-19 ICUs with those in 2 non–COVID-19 ICUs. Interviews with nurses and physicians who worked in the COVID-19 ICUs led us to hypothesize that COVID-19 ICUs have a higher alarm load, a higher number of specific alarm signals, and longer-sounding alarms than non–COVID-19 ICUs. We also hypothesized that staff working in COVID-19 ICUs experience more alarm fatigue than staff working in non–COVID-19 ICUs.

### There Was a Higher Alarm Load in the Provisional COVID-19 ICUs

COVID-19 ICUs had a significantly higher alarm load from red, yellow, and technical alarms. This higher alarm load led to an increased number of alarm flood conditions and concurrent alarm duration, escalating the nurses’ workload and potentially causing sensory overload [38]. While some differences can be attributed to the COVID-19 condition itself—such as the higher number of red critical alarms, possibly due to the high mortality rate and bad conditions of the patients—we suspect that most differences in alarm load were due to the interaction of staff with the alarm system (eg, not using the pause function when turning a patient from prone to supine position or vice versa, not adjusting thresholds specifically to patients’ conditions, not installing or not using monitoring profiles specific to patients with COVID-19 infection, or improperly applying sensors).

### Patients With COVID-19 Infection Had Similar Alarm Signals as Those Without COVID-19 Infection

We anticipated that some devices—such as ECG, NIBP, and IBP devices—would generate more alarms in COVID-19 ICUs due to the unique physiological manifestations of COVID-19, but this was not the case. Only red ECG alarms were notably more frequent in COVID-19 ICUs than in non–COVID-19 ICUs; other numbers of different clinical alarm signals were similar across all ICUs and seem to be a recurring theme because similar results were reported in previous studies [12,22].

In both ICU types, *ECG lead off* was the most frequent technical alarm signal. Interestingly, it occurred more than twice as often in COVID-19 ICUs. This also might be attributed to practices such as moving patients between prone and supine positioning, which necessitates the removal and subsequent reattachment of all sensors and electrodes each time.

### The Alarm Duration Was Equally Long in Both ICU Types

While our initial theory posited that alarm durations would be longer in COVID-19 ICUs—owing to the time required for staff to don protective equipment before entering a patient’s room—our findings did not confirm this hypothesis. We found no significant differences in the overall median alarm durations between COVID-19 and non–COVID-19 ICUs, except in the case of a few medical devices.

Devices displaying visual information such as ECG waveforms allow staff to rapidly assess alarm urgency. By contrast, the numerical values displayed by devices such as those measuring temperature or NIBP require more time for staff to interpret, potentially causing longer alarm durations.

We must also acknowledge the impact of other factors, for example, the floor layout of the unit, different unit policies [23], nurse-patient ratio [39], and the individual traits of the staff members [40].

Interestingly, red alarm durations were consistently longer than yellow alarm durations across all medical devices and in both ICU types. This may be due to health care providers promptly turning off yellow alarms first (without checking the patient’s condition) [39], placing more emphasis on critical alarms, and only then checking the patient’s condition.

In our exploratory analyses, we found that the alarm pause function was used more frequently in non–COVID-19 ICUs than in COVID-19 ICUs. This might be explained by the fact that many nurses in COVID-19 ICUs had limited critical care experience and therefore did not know that this function exists or how to use it; COVID-19 ICU staff also might have entered the patients’ rooms less often because they were required to don protective gear, which is a time-consuming process.

### ICU Staff Experienced More Alarm Fatigue in COVID-19 ICUs

While overall alarm fatigue questionnaire scores were similar between both types of ICUs, the situation varied among different health care professionals. Nurses and physicians in COVID-19 ICUs had significantly higher alarm fatigue scores, whereas support staff in both types of ICUs reported low alarm fatigue scores. Notably, a substantial portion of questionnaire participants in COVID-19 ICUs consisted of support staff, that is, students or nurses from normal wards (COVID-19 ICUs: 25/56, 45%; non–COVID-19 ICUs: 9/88, 10%), reflecting the recruitment of nurses from other services and students with varying levels of critical care experience due to the exceptional circumstances. Unlike the experienced and well-rehearsed teams typically found in non–COVID-19 ICUs, COVID-19 ICU teams often incorporated diverse teams that lacked experience in critical care medicine, training, and familiarity with the monitoring system and alarm management. This inexperience extended to their training and understanding of monitoring systems and alarm management, mirroring findings from existing literature [41]. In some hospitals, the patient-nurse ratio was increased, which occasionally resulted in poorer quality of care [42]. However, it is important to note that greater fatigue was identified among those working with patients with COVID-19 infection, rather than directly associating alarm fatigue with the COVID-19 condition itself.

There seemed to have been challenges with the alarm system in the COVID-19 ICUs that might have impeded appropriate monitoring. Due to the absence of central monitors in the corridors, dispensary room, and physicians’ room, it was difficult for health care providers to locate or swiftly identify and respond to alarms. Interviewees mentioned the protective gear worn in the COVID-19 ICUs as an additional burden when responding to alarms. Protective equipment can impede swift movement, thus slowing response times to alarms, which might intensify the stress and sensory overload associated with alarm fatigue. This finding aligns with the conclusions drawn by Akturan et al [43] that suggested that personal protective equipment could contribute to increased alarm fatigue.

### Recommendations for ICU Alarm Systems in Future Pandemics

Due to extensive international traffic, the risk of future pandemics remains high in a globalized world [44], and the COVID-19 pandemic will be followed by a new pandemic at some point. Such pandemics will again likely require the setting up of provisional ICUs to cope with rapid patient admissions. When preparing for such events, we recommend also preparing the alarm systems and their human operators. Even outside of pandemics, the shortage of specialist staff is increasing the willingness of authorities to deploy untrained personnel in certain functions in the medical field.

Alarm management aims to reduce the number of unnecessary alarms on the premise that a lower alarm rate decreases alarm fatigue among staff [22]. Provisional ICUs can benefit from effective alarm management, as do regular ICUs, where it helps to significantly decrease the number of false alarms and the overall alarm burden [45-49].

Unnecessary alarms stem from insufficient alarm management knowledge, including using default instead of customized alarm limits, rarely using the pause function during patient manipulation or being unaware of its existence, using insufficient consumables, or attaching electrodes improperly [50]. Previous studies [51] highlight a lack of knowledge about these functions among nurses, pointing to a pressing need for education on physiological monitors [52].

**Figure 1:**
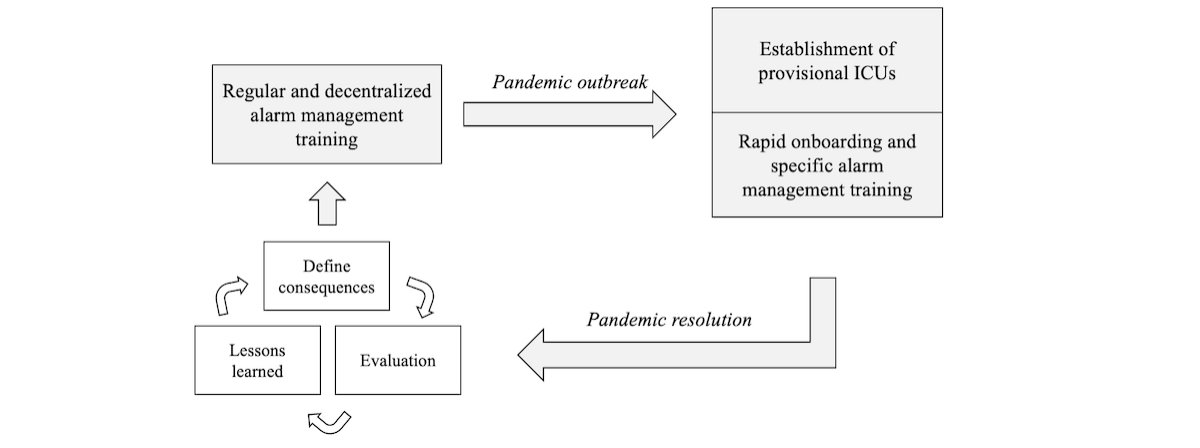
Two-phase approach to intensive care unit (ICU) alarm system preparedness and refinement. This figure details continuous training during nonpandemic periods, rapid onboarding, and specialized training at the onset of a pandemic, followed by an evaluation cycle after the pandemic to improve future alarm management strategies.

Given the urgency with which new staff members have to be onboarded, the training should be decentralized and easily accessible. To cater to these requirements, we recommend developing educational microcredentials, such as video tutorials that provide guidance on adjusting alarm limits on specific monitoring devices, implementing remote alarm turn-off, and transitioning from individual to smart alarm systems. To visualize and operationalize our approach to pandemic preparedness in ICU alarm systems, we propose a bicyclical strategy, as illustrated in Figure 1. This strategy underscores the significance of continuous learning and adaptability in alarm management. Operating modern bedside monitors requires a blend of cognitive knowledge, psychomotor skills, critical thinking, and an understanding of alarm systems [53]. Hence, this educational program should use the skillmap from the study by Sowan et al [51] as a foundation, expanding it to address the broader technical and practical aspects of monitor use. Modular content that teaches specific skills can be easily adapted to meet unique situational needs; for example, tutorials can be designed to explain how to respond to alarms while wearing protective gear or how to mobilize patients in prone position without causing alarm artifacts. These resources could be beneficial for ICU staff training not only during pandemics but also in routine situations.

### Limitations

ICUs function as complex sociotechnical systems, making it inherently challenging to compare them. All ICUs were equipped with similar technical equipment (eg, mechanical ventilators and dialysis devices), but our comparison between ICUs was complicated by the variations in the specific devices used across units, leading to differences in alarm signals. Therefore, we only included alarms that occurred in all units. In addition, numerous devices in all ICUs are not connected to the central monitoring system, meaning that the alarms could not be recorded and evaluated. Alarms from mechanical ventilation had to be excluded from the ICU data due to a technical error, preventing us from testing our hypothesis about an increase in such alarms in COVID-19 ICUs.

The monitoring systems in the 2 ICU types varied in their version numbers and settings, further affecting the comparability. In COVID-19 ICUs, the *ECG lead off* alarm was set as a yellow alarm, while in non–COVID-19 ICUs, it was set as a blue alarm. This discrepancy likely stemmed from a lack of awareness or understanding about the settings. We had to exclude this alarm from the yellow alarm load comparison due to this discrepancy.

Our metrics, calculated relative to the number of occupied beds per day, could potentially skew the results. When metrics are calculated this way, it might not accurately reflect the real-life impact of alarm flood conditions on health care workers. In a larger ICU (such as the one with 24 beds), the same rate of alarms per bed would create a larger absolute number of alarms because there are more beds. Consequently, the staff in this ICU would be exposed to a higher number of total alarms than the staff in a smaller ICU (one with, say, 10 beds), although the rate of alarms per bed is the same. While the surgical and medical ICUs were approximately fully occupied over the entire period under study, the occupancy of the COVID-19 ICUs often varied, depending on the patient load. The average bed occupancy was 97.26% and 92.27% in the surgical and medical ICUs, respectively; and 88.79% and 82.75% in COVID-19 ICU-A and COVID-19 ICU-B, respectively. The occupancy of all examined ICUs is reported in Figure S2 in Multimedia Appendix 1. The staff members interviewed from the COVID-19 ICUs were not the same as those from the non–COVID-19 ICUs, which introduces a limitation related to team differences and experience levels.

Regarding the alarm durations, we could not investigate the impact of the isolation process by analyzing alarm response times between COVID-19 ICUs and isolation rooms in non–COVID-19 ICUs. Due to the provisional and time-limited nature of the COVID-19 ICUs, the alarm fatigue in health care providers might reflect not only the conditions in the COVID-19 ICUs but also the individual exposure to alarm load during their former career. We cannot definitively determine whether the increased alarm load was caused by the COVID-19 condition itself or by the differences in ICU settings. Unfortunately, more detailed clinical data for every patient associated with the alarm data were not available at the time of the study. From a clinical perspective, the patient cohorts in the COVID-19 ICUs and general ICUs were comparable in terms of the severity of their conditions. In addition, fatigue among health care professionals working with patients with COVID-19 infection was inherently greater due to the overall stress and workload of managing COVID-19 cases, which cannot be attributed solely to alarm load. We suggest propensity score matching or similar statistical techniques as areas for future research. Similarly, determining isolation rooms from non–COVID-19 ICUs is recommended for further studies to gain a deeper understanding of the impact of the isolation process on alarm response times.

### Conclusions

In this study, the COVID-19 ICUs registered significantly more alarms than the non–COVID-19 ICUs. The higher number of alarms led to a higher level of alarm fatigue among the clinicians working in COVID-19 ICUs. We believe that this was caused by the high proportion of untrained staff who were deployed to the temporary ICUs during the pandemic and the provisional setting. The absence of central monitors in individual rooms and corridors further compounded these challenges, making it difficult for health care providers to swiftly identify and respond to alarms. However, it is important to note that our findings are limited by the study design and specific circumstances during the pandemic, which might affect the strength of our conclusions. Further studies are warranted to better understand the broader implications of alarm management in different ICU settings.

To mitigate alarm overload in provisional ICUs during future pandemics, we recommend creating skill-oriented video tutorials on alarm management and monitor use. These tutorials should provide easily accessible training for new staff, who may be rapidly recruited and could have limited or no prior ICU experience. This educational material could equip them with the necessary knowledge to effectively navigate the ICU alarm system.

## Appendix

Multimedia Appendix 1 Alarm load and management differences between COVID-19 and non–COVID-19 intensive care units (ICUs). Figures include alarm frequencies by device and color, unit occupancy, alarm durations, and adjustments in settings across ICUs. Table data cover interview questions, alarm log structure, included alarm signals, hypothesis testing group sizes, and statistical analysis of alarm load per bed per day.

## Abbreviations

ECG: electrocardiogram
IBP: invasive blood pressure
ICU: intensive care unit
NIBP: noninvasive blood pressure
REDCap: Research Electronic Data Capture
SpO2: peripheral oxygen saturation

## Data Availability

The data sets generated and analyzed during this study are available in the Zenodo open data repository [54]. The code is available from the corresponding author on reasonable request.
