# Supplementary material for: Alarm Management in Provisional COVID-19 Intensive Care Units: Retrospective Analysis and Recommendations for Future Pandemics
Source: JMIR Med Inform. 2024 Sep 9;12:e58347. doi: 10.2196/58347 (PMC11420579; doi:10.2196/58347)
Supplement: Multimedia Appendix 1 [file medinform_v12i1e58347_app1.docx]

# Supplementary Material - Figures


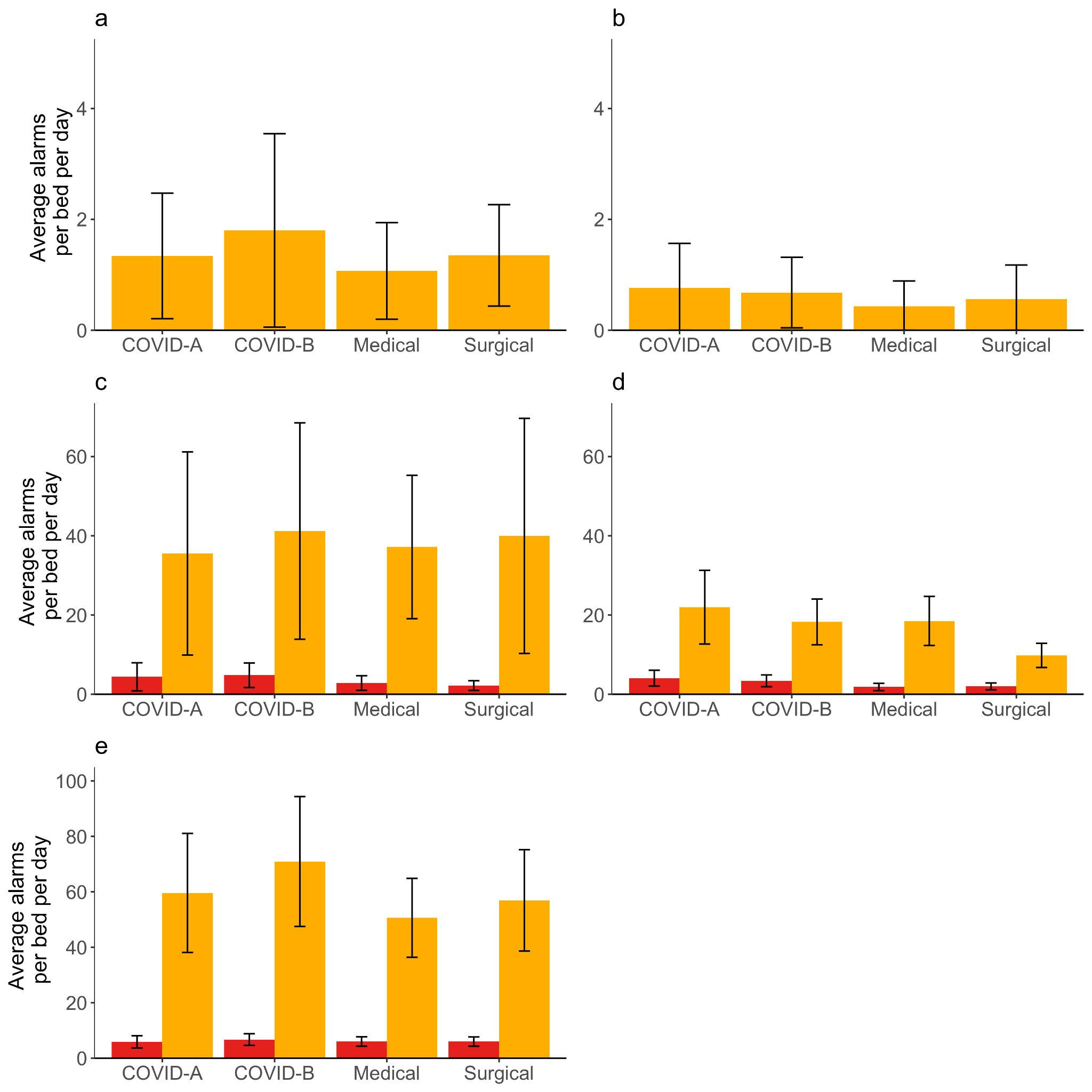


**Fig. 1 Average clinical alarm load subdivided by alarm color (red and yellow alarms) and medical device of all ICUs and ICUs.** Bars represent mean (SD). **a** Alarms caused by temperature device **b** Alarms caused by Non-Invasive Blood Pressure device (NIBP) **c** Alarms from ECG device **d** alarms from SpO2 device **e** Alarms from IBP device. The average number of alarms from yellow alarms differs between COVID-19-19 and non-COVID-19-19 ICUs. While the average number of red alarms is similar within the ICU types, it differs between them.

The IBP device was responsible for most alarms, followed by the ECG and SpO2 device (Figure 1). In COVID-19 ICUs, more yellow alarms were issued from IBP, ECG, NIBP, and the temperature device. Red alarms from the ECG and SpO2 devices occurred more than twice as often in the COVID-19 ICUs. All red alarms from the SpO2 devices are caused by the *Desat* alarm signal. The differences in the alarm load of red alarms from the ECG device are caused by the different alarm frequencies of the *VTachy* and *xTachy* alarm signals *(VTachy:* mean_COVID-19-A_: 1.47 (SD 2.17), mean_COVID-19-B_: 0.88 (SD 1.03), mean_Surgical_: 0.52 (SD 0.62), mean_Medical_: 0.41 (SD 0.68); *xTachy:* mean_COVID-19-A_: 2.22 (SD 2.51), mean_COVID-19-B_: 3.21 (SD 2.59), mean_Surgical_: 0.95 (SD 0.82), mean_Medical_: 1.34 (SD 1.36).

The staff in COVID-19 ICUs used the alarm pause function less often (5.08/12.21; Figure 5). The medical ICU has the highest proper pause to pause ratio of 0.08, followed by the surgical ICU and COVID-19-A with 0.04. COVID-19-B has the lowest value of 0.03. Health providers used threshold changes less often in COVID-19 units (median_COVID-19_: 1.75; median_non-COVID-19_: 3.80). The number of profile changes per bed per day varies across all ICUs but was lower in COVID-19 ICUs (median_COVID-19_: 0.06, median_non-COVID-19_: 0.10; Figure 5).

**
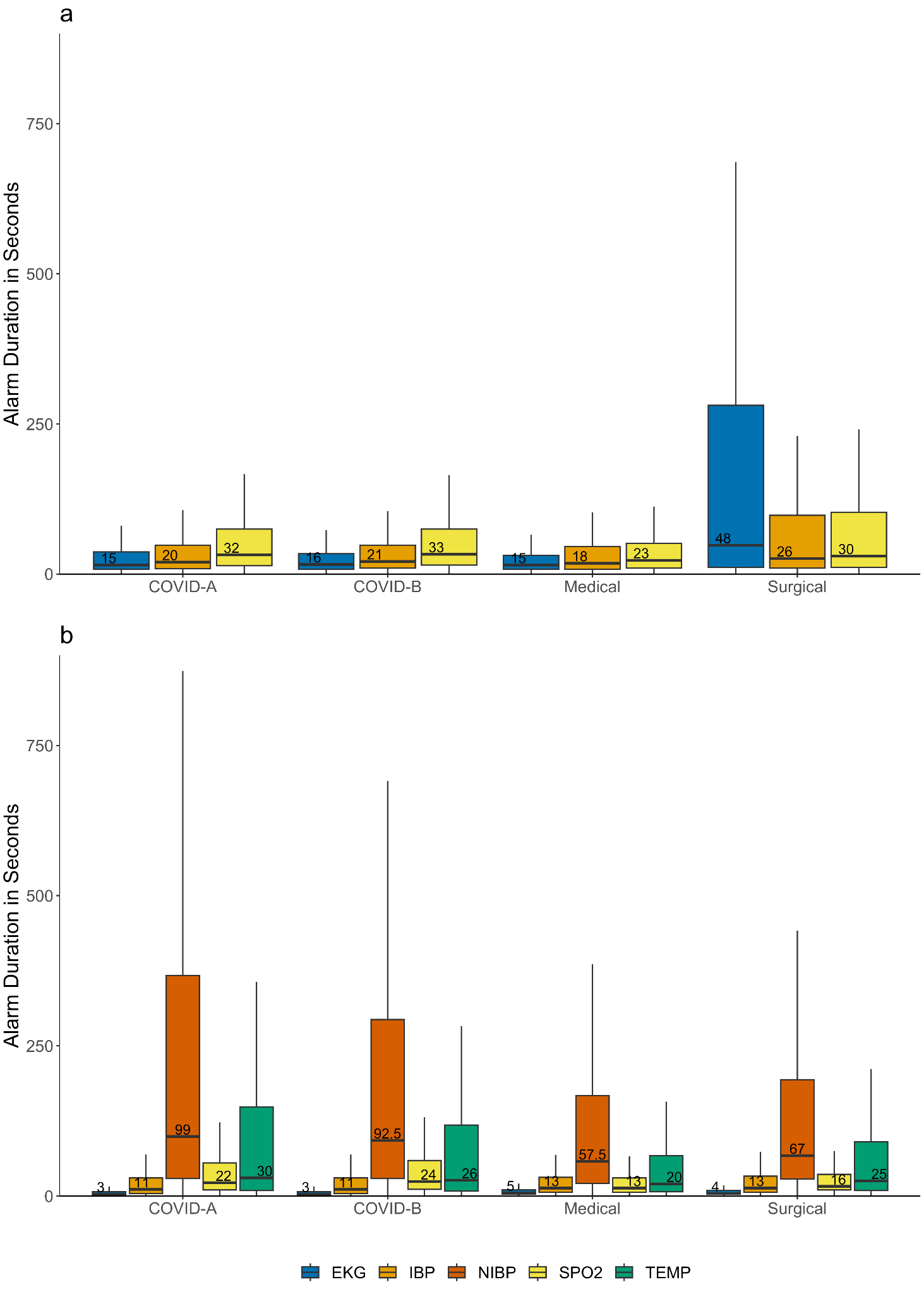
**

**Fig. 2 Boxplots of median alarm durations of clinical alarm durations splitted by medical device and alarm color. a** Alarm durations caused by red alarms. **b** Alarm durations caused by yellow alarms. Alarm durations from red alarms are similar across Medical and COVID-19-A and B, while they differ in Surgical. All alarm durations from red alarms are longer than alarm durations from yellow alarms from the corresponding medical devices.


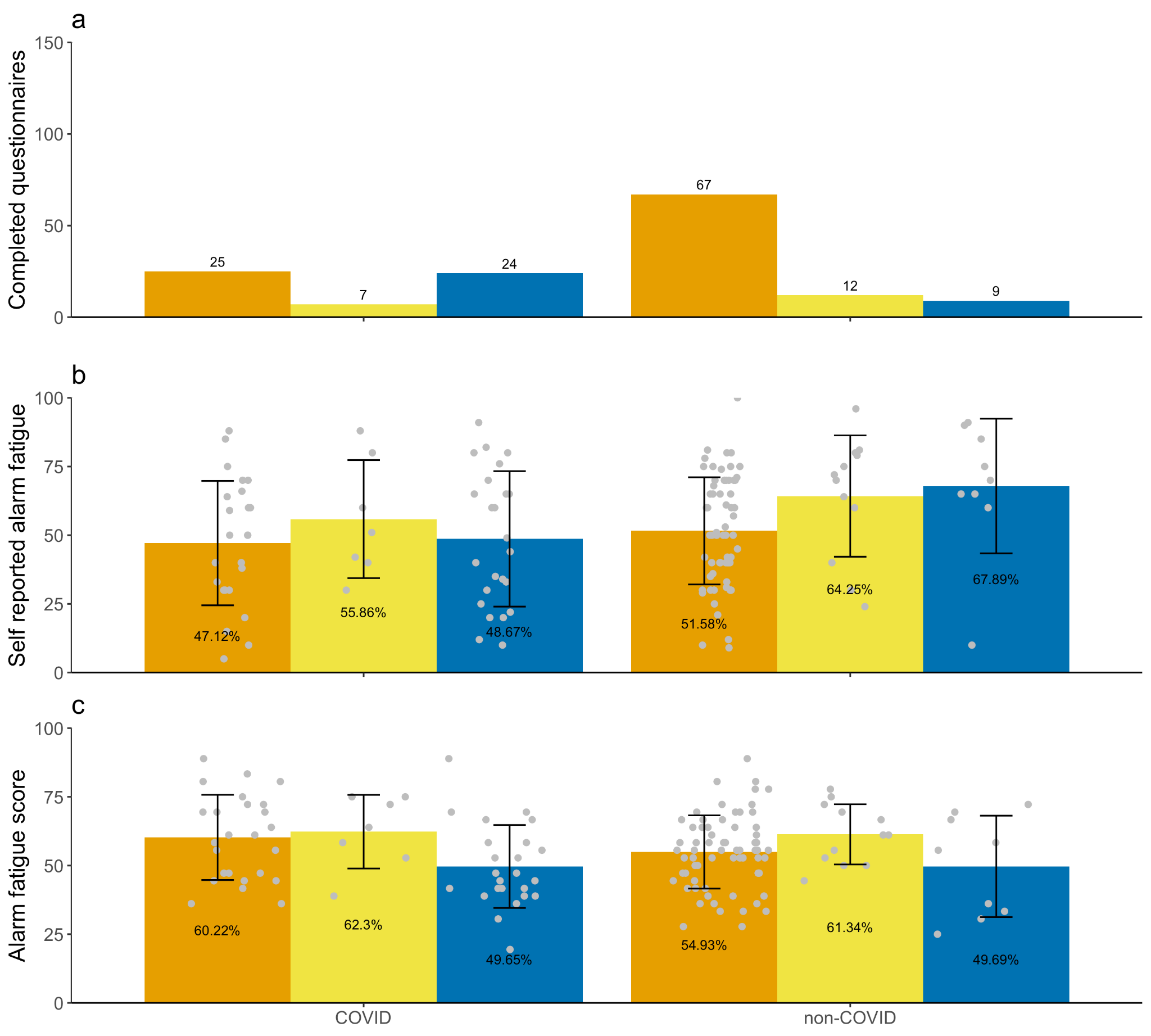


**Fig. 3. Results of the alarm fatigue questionnaire subdivided by ICU type and staff profession. a** Completed questionnaires. **b** Self-reported alarm fatigue. The values displayed are the average reported alarm fatigue **c** Results of the alarm fatigue score. The displayed values are the average alarm fatigue score. Nurses are displayed in orange, physicians in yellow, and support staff in blue. Nurses and physicians in COVID-19 ICUs had a higher alarm fatigue score but a lower self-reported alarm fatigue.


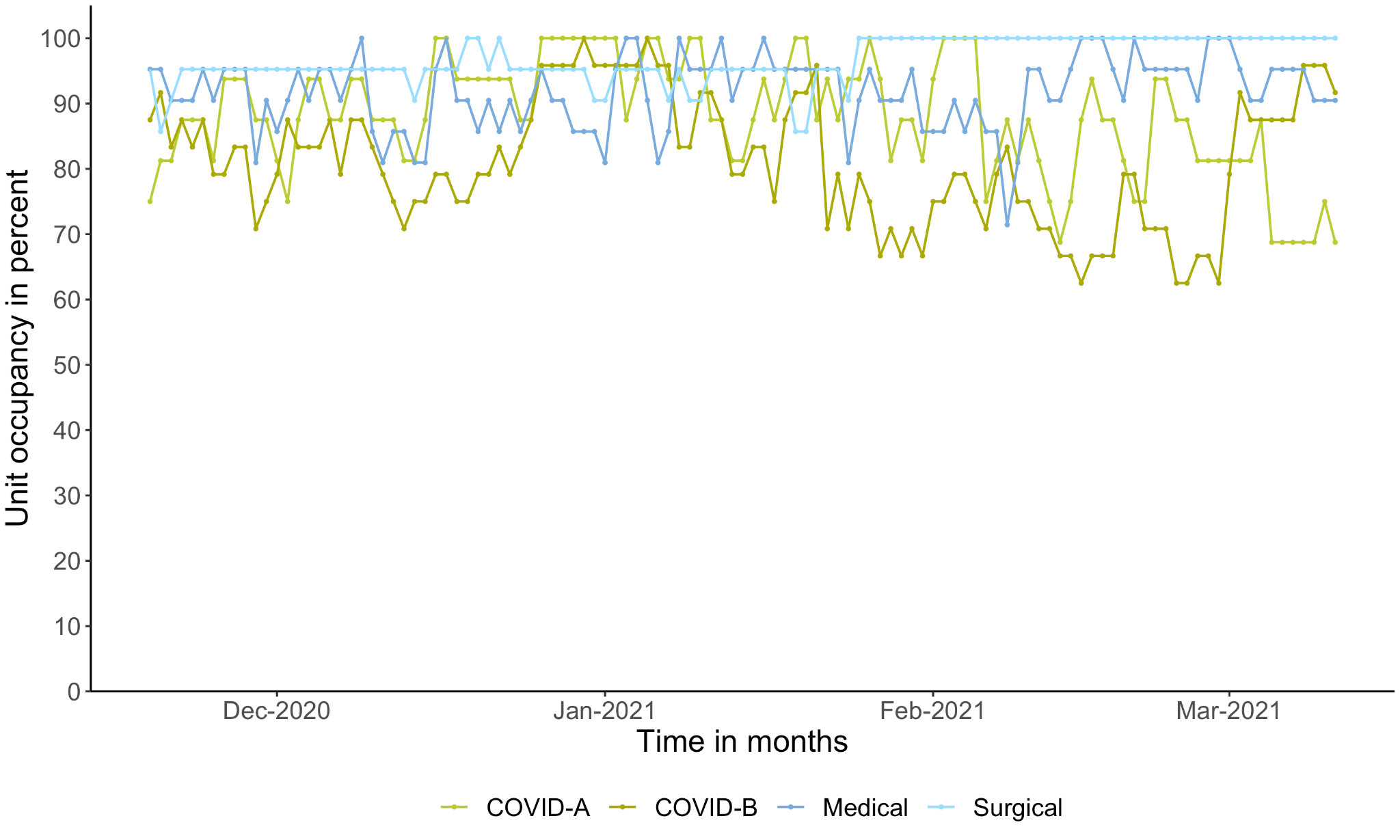


**Fig. 4. Figure Unit occupancy of all examined ICUs and subunits.** Non-COVID-19 ICUs had a higher unit occupation than COVID-19 ICUs.

**
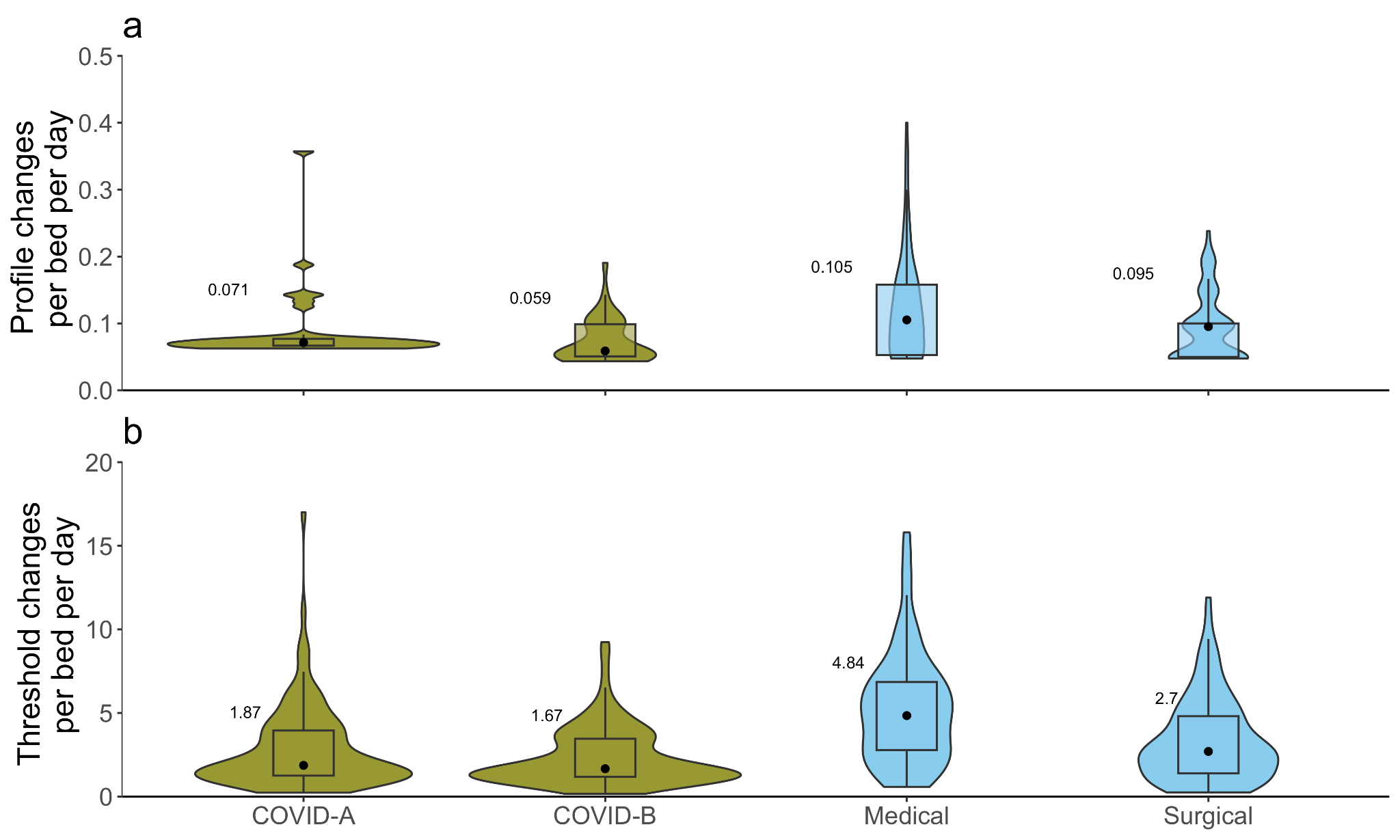
**

**Fig. 5 Violin plots of threshold changes and profile changes per bed per day from all ICUs. a** Profile changes per bed per day **b** Threshold changes per bed per day. In the medical ICU, profiles and thresholds were adjusted the most often. Results from COVID-19 ICUs are colored in olive, non-COVID-19 ICUs in cyan. All distributions are heavily skewed to the right; therefore, values are reported in median.

# Supplementary Material - Tables

**Table 1.** Interview Questions, translated from German to English.

| No. | Interview Question |
| --- | --- |
| 1 | What are the biggest differences in the alarm situation in COVID-19 ICUs compared to non-COVID-19 ICUs? |
| 2 | Which medical device could cause more alarms and do certain alarm signals occur more often in COVID-19 ICUs? |
| 3 | Do certain COVID-19-specific treatments causes more alarms? |
| 4 | Do certain COVID-19-specific treatments causes more alarms? |
| 5 | Which additional stressors exist in COVID-19 ICUs? |
| 6 | What percentage of staff had no or little critical care experience? |
| 7 | Did inexperienced staff respond inadequately to alarms? |
| 8 | Did the unit culture in COVID-19 ICUs have an influence on alarm management? |
| 9 | Does the personal protection equipment have an influence on the usability of monitoring devices? |
| 10 | Does the unit layout from the provisional COVID-19 ICUs differ from non-COVID-19 ICUs? |
| 11 | How do patients in COVID-19 ICUs differ from patients in non-COVID-19 ICUs and does that have a influence on the alarm situation? |

**Table 2.** Structure of the cleaned alarm log dataframes.

| Column | Description | Datatype |
| --- | --- | --- |
| Date | day and time of log entry | Date |
| Aktion | Description of the occuring event | character |
| Bed | bed where the event occured | character |
| Unit | Unit in which event occured | character |
| total beds | total amount of beds | numeric |
| Shift | Shift (early, late, night, night.early, early.late, late.night) | character |
| Alarm Color | alarm color (blue, yellow, red) | character |
| Alarm Start Time Alarm end time | Start and Endtime of the alarm, extracted from Aktion | POSIXt |
| alarm type | type of alarm that occured | factor |
| value measured, | measured value of the vital parameter | numeric |
| alarm threshold | threshold that was used when alarm occured | numeric |
| threshold excess | value how much the measured value overshoots the threshold | numeric |
| alarm group | medical device that generated the alarm | character |
| pause duration | how long the pause lasted in seconds | numeric |
| alarm duration | how long the alarm lasted in seconds | numeric |
| Patient ID | ID of the patient that triggered the event | integer64 |
| Admission and discharge date | Date of admission and discharge | POSIXt |
| year of birth | year of birth of the patient | numeric |

## **Table 3.** Included alarm signals.

|  | yellow alarms | red alarms | blue alarms |
| --- | --- | --- | --- |
| NIBP | NBPm, NBPs | - | - |
| TEMP | TBlut, TKern, Temp, THaut | - | - |
| SPO2 | Puls, SpO2.low, | Desat | - |
| IBP | ABPm, ABPs.high, ABPs.low, ABPd.high, ABPd.low, ABPm.high, ABPm.low | ABP Bereich?, ABPd, ABPm, ABPs, ABPunterbrochn, ART Bereich?, ARTunterbrochn | - |
| ECG | AF.high, AF.low, HF.high, HF.low, Multiform VES, Paroxysmale VT, VES-Paar | VTachy, xBrady, xTachy, Asystolie, Brady/P,  Vent Fib/Tachy | - |
| Technical alarms | ABP Bereich?, P Bereich?, ZVD Bereich?, ART Bereich? | Pat.-ID ueberpruef, EKG-Elektrdn ab | EKG Elektrdn ab,  ARTunterbrochn |

**Table 4:** Alarm load in alarms per bed per day and statistical analysis from all ICUs subdivided in medical device and alarm color. The average alarm load of all devices and colors was calculated in alarms per bed per day and reported in mean (SD).

|  | Surgical | Medical | COVID-19-A | COVID-19-B |
| --- | --- | --- | --- | --- |
| ECG (yellow) | 40.0 (SD 29.7) | 37.2 (SD 18.1) | 42.7 (SD 25.9) | 48.9 (SD 27.4) |
| ECG (red) | 2.18 (SD 1.23) | 2.84 (SD 1.85) | 4.39 (SD 3.56) | 4.80 (SD 3.10 ) |
| IBP (yellow) | 56.9 (SD 18.3) | 50.6 (SD 14.2 ) | 59.6 (SD 21.5) | 71.0 (SD 23.4) |
| IBP (red) | 6.98 (SD 1.67) | 6.80 (SD 1.76) | 7.03 (SD 2.38) | 8.00 (SD 2.20) |
| NIBP | 0.559 (SD 0.616) | 0.430 (SD 0.458) | 0.765 (SD 0.80) | 0.675 (SD 0.638) |

## **Table 5.** Group Sizes of Hypothesis testing

| Sample Size | Sample Size non-COVID-19 | Sample Size COVID-19 |
| --- | --- | --- |
| Technical alarms | 20,294 | 37,109 |
| NIBP | 1,882 | 2,275 |
| Temperature | 5,449 | 6,220 |
| SpO2(yellow) | 62,923 | 75,387 |
| SpO2 (red) | 8,524 | 13,911 |
| IBP (yellow) | 241,620 | 252,096 |
| IBP (red) | 26,967 | 24,359 |
| ECG (yellow) | 173,730 | 147,629 |
| ECG (red) | 17,573 | 11,211 |
